# Household Food Insecurity, Dietary Diversity, and Stunting in Sub-Saharan Africa: A Systematic Review

**DOI:** 10.3390/nu13124401

**Published:** 2021-12-09

**Authors:** Goudja Gassara, Jihua Chen

**Affiliations:** Department of Nutrition Science and Food Hygiene, Xiangya School of Public Health, Central South University, Changsha 410008, China; goudjag@yahoo.fr

**Keywords:** dietary diversity, food insecurity, malnutrition, public health, stunting, Sub-Saharan Africa, systematic review, undernutrition

## Abstract

Background: The significant public health problem in Sub-Saharan Africa of household food insecurity is an underlying cause of malnutrition in Sub-Saharan Africa. This study aims to systematically study the association between household food insecurity, dietary diversity, and stunting. Methods: This review was carried out based on the recommendations of PRISMA (2015). We searched the literature in six bibliographic databases: PubMed, EMBASE, Science Direct, Web of Science, Google Scholar, and Scopus. The research was based on studies conducted in Sub-Saharan Africa about household food insecurity, dietary diversity, and stunting and was published between 2009 and 2020. Results: Out of 2398 original articles identified, only 21 articles met the specific requirements of this review. Two-thirds of the articles selected showed that stunting was linked to household food insecurity and dietary diversity. Conclusions: This study found that household food insecurity and dietary diversity are significantly associated with stunting in Sub-Saharan Africa. This review recommends that in order to yield a sustainable fight against childhood malnutrition in Sub-Saharan Africa, reliable guidelines and strategies are needed to address these factors related to malnutrition.

## 1. Introduction

Household food insecurity (HFI) is defined as the lack of physical, social, and economic access to sufficient, safe, and nutritious food to meet the needs and food preferences for an active and healthy life [1]. As the matter stands, and in developing countries, household food insecurity frequently manifests itself in the form of poorly diversified and micronutrient-deficient diets. Currently, HFI is associated with poor dietary diversity, poverty, and malnutrition, putting children’s development at risk [2].

Food and nutritional insecurity are observed in two situations: insufficient food (hunger and malnutrition) and in cases of diseases resulting from inadequate nutrition (excess weight and specific needs). When these situations arise, it is up to the health sector to provide adequate treatment, rehabilitation, and adequate prevention to avoid the occurrence of other complications [3,4,5].

Despite favorable global economic growth, food insecurity and malnutrition continue to cause suffering to many of the world’s population, particularly in Africa [6,7].

Household food insecurity, racial disparities, poverty, neglect, and family stress are seen as risk factors for early childhood development (ECD) [8]. A few studies have shown the association between household food insecurity (HFI) and poor early childhood development (ECD) [9,10,11]. One study showed that HFI was associated with developmental risk and adverse effects on function, academic performance, and social skills development in preschool and school-aged children in the United States (USA) [9]. In the USA, another study described HFI as associated with reduced performance, especially in academic, social, and psycho-emotional fields, from infancy through adolescence [10].

Achieving food security remains an important challenge in Sub-Saharan Africa. There is a growing gap between consumption, nutrition, and food availability at regional, household, and individual levels. Almost half of the population of this region lives below the poverty line and depends on rain-fed agriculture, animal husbandry, and fishing for their survival. Thus, to ensure its food security, Africa must considerably increase its agricultural production and productivity. This assertion is confirmed by a study which shows that despite the favorable developments in food security throughout the world, the problem remains very worrying in SSA, where 180 million people were undernourished from 1995–1997, that is to say, a third of population [12].

In Sub-Saharan Africa, food insecurity is due to conflict, climate change, and economic problems. Overall, progress in the fight against malnutrition remains insufficient in Africa. Three quarters of Africans cannot afford a healthy diet according to the Food and Agriculture Organization (FAO). Thus, more than half cannot afford an adequate supply of nutrients [13].

Defined as the number of different food groups consumed during a given period by an individual or household, dietary diversity in many situations is an important indicator of the nutritional status [14,15]. Several studies have shown that low nutritional variety is often associated with growth retardation in children [16].

During the critical phase of a child’s development, i.e., from conception until the first 24 to 59 months after birth, stunting can lead to delayed cognitive and physical development that is difficult to reverse [17,18,19]. Therefore, in the short and long term, malnutrition has adverse effects on children’s health and negatively affects the economic productivity of nations [20]. In Sub-Saharan Africa, most children under five suffer from stunting (39%) [21].

No previous systematic review analyzed the relationship between household food insecurity and stunting and the relationship between dietary diversity and stunting in children under 5 in Sub-Saharan Africa. This study aimed to examine the relationship between these different factors, thus allowing the establishment of guidelines and practical interventions to improve the population’s standard of living and thus reduce the prevalence of malnutrition in the region and sub-regions.

## 2. Materials and Methods

### 2.1. Household Food Insecurity Indicator

For this systematic review, the food security indicator used is the Household Food Insecurity Access Scale (HFIAS). The HFIAS scale consists of nine occurrence questions and nine frequency of occurrence questions.

### 2.2. Anthropometric Indicator

The anthropometric indicator retained for this systematic review is stunting. Nutritional indices in the articles were calculated using the WHO Anthro 3.1.0. The results were classified according to the thresholds of the World Organization Health 2006 [22]. Children with HAZ score below -2SD (standard deviation) were stunted. Children with HAZ scores greater than or equal to -2SD were considered normal.

### 2.3. Food Diversity Indicators

For this systematic review, the indicators used are three in number: the 24 h reminder, the 7-day reminder, and the standard 48 h reminder technique [23]. The reference for the dietary diversity period may vary, but most often corresponds to the day (24 h reminder) or the previous week (7-day reminder) [24,25].

✓ For 24 h reminder, the household dietary diversity score (HDDS) equals the total number of different food groups consumed in the last 24 h by a household member at home, including foods prepared at home but eaten outside, such as a lunch bag [26,27].
✓ Concerning the 7-day reminder, this corresponds to the number of food groups consumed by a member of the household member during the last seven days from the total of 13 food groups.
✓ Finally, the 48 h reminder corresponds to all food and drinks consumed by household members day and night during the previous 48 h at different times of meals [23].

### 2.4. Research Strategy

This systematic review of the literature was carried out in pairs according to the PRISMA recommendations (Preferred Reporting Items for Systematic reviews and Meta-Analyses) of 2015 [28]. The search was carried out based on a list of keywords. Subheadings of keywords were to exhaustively search for articles from six bibliographic databases: PubMed, EMBASE, Science Direct, Web of Science, Google Scholar, and Scopus. The research focused on studies conducted in all countries (48) of Sub-Saharan Africa and published between 2009 and 2020. We have searched for additional relevant studies manually using the retrieved article references. The following combined keywords were used for the search:

(Household food insecurity* OR HFI* OR dietary diversity* OR DD* OR stunting* OR stunt*).

### 2.5. Inclusion and Exclusion Criteria

The inclusion criteria were: (1) original articles related to the topic of interest of this study; (2) studies evaluating the association between household food insecurity and stunting as well as dietary diversity and stunting in children under five; (3) studies carried out in Sub-Saharan Africa; (4) studies published between 2009 and 2020; (5) cross-sectional studies (books, qualitative studies, policy briefs, case studies, and theses were excluded); (6) studies published in a peer-reviewed journal (reviews, non-peer-reviewed research, and comments were excluded); (7) studies published in English.

### 2.6. Data Extraction

The use of the EndNote library allowed us to remove all duplicate articles. From the title and the summary, the first reading phase allowed us to remove some selected articles. Then after a full reading of the articles, we selected studies that met the inclusion/exclusion criteria. We independently reviewed the retained articles and rejected all those that met the exclusion criteria. The following information was extracted from the articles selected: author(s), year, country, number of children, age of children, number of households, sample, location, study purpose, method of HFI evaluation, method of DD evaluation, stunting, and finally the results (HFI/Stunting, DD/Stunting, and Prevalence Stunting) (Table 1). The studies were also grouped by country into SSA sub-regions (West Africa, East Africa, Southern Africa, and Central Africa) based on the United Nations (UN) scheme classification and by year of publication.

### 2.7. Quality Assessment

The Joanna Briggs Institute (JBI) checklist for Cross-Sectional Analytical Studies [29] was applied to assess the quality of the articles reviewed. The assessment of the quality of the selected studies is presented in the Supplementary Table S1. The selected studies were homogeneous, and all analyses were of high quality according to the JBI-MAStARI.

**Table 1:** Summary of selected articles.

West Africa						
Author [Ref.], Year, Country	No. of Children (*N0*), Age, No. of Households (*N1*)	Sample/Location	Study Purpose	Methods of HFI, DD Evaluation and Stunting	HFI and Stunting	DDS and Stunting
Hatley, A. et al. [30] 2000 Mali	*N0* = 526 Urban and 1789 Rural 6–59 months *N1* = 329 Urban and 488 Rural	The study was conducted in Koutia County, Sikasso region. The sample consists of 329 urban households and 488 rural households with 526 urban children and 1789 rural children aged 6 to 59 months.	To analyze the associations between the food variety score (FVS), the dietary diversity score (DDS), and children’s nutritional status.	-Socioeconomic and demographic questionnaires -24 h reminder -HAZ		Doubled risk of stunting in urban children with low dietary diversity score.
Mahama, S. et al. [31] 2013 Ghana	*N_0_* = 337 6–36 months *N_1_* = 337	The study was conducted in the metropolis of Tamale. A sample of 337 mother/child pairs made up the study.	Assess the extent of household food insecurity and its consequences on the nutritional status of children aged 6 to 36 months and their mothers.	-Socioeconomic and demographic questionnaires -HFIAS -7-day reminder -HAZ	The relationship between food insecurity and stunting was stronger in children aged at least 23 months (*p* = 0.019). 29.8% stunting. 54% household food insecurity.	
Ali, S. et al. [32] 2018 Burkina Faso	*N_0_* = 251 6–59 months *N_1_* = 251	The study was carried out in the sub-Sahelian villages of Kamadena and Dara. A total of 251 children were included in the study.	Evaluate dietary diversity patterns in children under 5 years of age during the lean season (July) and assess the association between dietary diversity and nutritional status.	-Sociodemographic questionnaires -7-day reminder -HAZ		The association between dietary diversity and linear growth was stronger in children younger than 36 months. Stunting 27.7% in Kamadena against 13.1% in Dara.
**Central Africa**						
**Author** **[Ref.],** **Year,** **Country**	**No. of Children (*N0*),** **Age,** **No. of Households (*N1*)**	**Sample/Location**	**Study Purpose**	**Methods of HFI, DD Evaluation and Stunting**	**HFI and Stunting**	**DDS and Stunting**
B.N. Ekesa et al. [33] 2011 DRC* Burundi * DRC = Democratic Republic of Congo	*N_0_* = 281 24–59 months *N_1_* = 281	The study was carried out in the Gitega area and the Butembo area. The sample includes 281 households, including 138 in Butembo and 143 in Gitega.	Assess the dietary diversity and nutritional status of preschool children.	-Sociodemographic questionnaires -24 h reminder -HAZ		DRC: 78.86% stunting, no relationship between dietary diversity and stunting (*p* > 0.05). Burundi: 63.57% stunting. In Burundi, the relationship between dietary diversity and weight for age was not significant (r^2^ = 0.051).
**East Africa**						
**Author** **[Ref.],** **Year,** **Country**	**No. of Children (*N0*),** **Age,** **No. of Households (*N1*)**	**Sample/Location**	**Study Purpose**	**Methods of HFI, DD Evaluation and Stunting**	**HFI and Stunting**	**DDS and Stunting**
Ali, D. et al. [34] 2013 Ethiopia	*N_0_* = 2356 6–59 months *N_1_* = 2992	The baseline survey in Ethiopia included 2992 households and 2356 children aged 6–59 months; surveys conducted in 75 enumeration areas in the 2 regions.	Examine the association between HFI and child undernutrition in Bangladesh, Ethiopia and Vietnam using HFIAS.	-Sociodemographic questionnaires -HFIAS -24 h reminder -HAZ		50.7% stunting in children. HFI was significantly associated with stunting.
Zipporah, N. et al. [35] 2014 Kenya	*N_0_* = 277 6–36 months *N_1_* = 277	A sample of 277 women−child couples was selected. The study was conducted in two counties (Machakos and Makueni).	Contribute to improving food security for women and men in communities prone to hunger.	-Sociodemographic questionnaires -24 h reminder -HAZ		There is a significant association (*p* = 0.023) between severe food insecurity and the nutritional status of children. 33.8% stunting.
Motbainor, A. et al. [36] 2015 Ethiopia	*N_0_* = 3964 0–59 months *N_1_* = 4110	The sample is made up of 4110 households chosen at random. The study was carried out in the Gojjam East and West areas of the Amhara region.	Determine the level of child malnutrition by comparing the two populations and assess the association between food insecurity and the nutritional status of children.	-Sociodemographic questionnaires -HFIAS -24 h reminder -HAZ	Food insecurity was significantly associated with wasting (*p* < 0.05). 37.6% stunting.	Dietary diversity associated considerably with stunting (*p* < 0.05).
Mutisya, M. et al. [37] 2015 Kenya	*N_0_* = 6858 6–23 months *N_1_* = 6552	The sample is made up of 6858 children and 6552 households. The study was carried out in two informal settlements (Viwandani and Korogocho) in the capital Nairobi.	To examine the effect of household food security status and its interaction with household wealth status on stunting in children aged 6 to 23 months.	-Socio-economic and demographic questionnaires -HFIAS -HAZ	According to the three Cox regression models, this study shows that household food security is significantly associated with stunting. 49% stunting.	
M’Kaibi, F.K. et al. [38] 2016 Kenya	*N_0_* = 500 24–59 months *N_1_* = 500	The study was conducted in two rural areas (Akithii and Uringu). The sample is composed of 500 respondents.	To determine the role of dietary diversity (DD), household food security (HFS), and agricultural biodiversity (AB) on stunting in children.	-Sociodemographic questionnaires -HFIAS -24 h reminder -HAZ	There is no significant relationship between household food security and the nutritional status of children.	There is a significant positive relationship in this study between stunting in children and the dietary diversity of the child in phase 2 of the study. The percentage stunting for phase 1 was 34.7% in Akithii and 26.3% in Uringu.
Abdurahman, A.A. et al. [39] 2016 Ethiopia	*N_0_* = 453 24–59 months *N_1_* = 453	The total sample is made up of 453 households. The study was carried out in the Eastern Haraghe area of Haromaya district.	This study aimed to examine the association between household food insecurity and the nutritional status of children aged 24 to 59 months.	-Socio-economic and demographic questionnaires -HFIAS -HAZ	Household food insecurity was significantly predictive of being underweight (*p* = 0.05). 61.1% stunting.	
Mulu, E. et al. [40] 2017 Ethiopia	*N_0_* = 555 6–59 months *N_1_* = 576	The sample is made up of 576 households with children under five. The study was carried out in the Sekela district.	This study was conducted to compare the nutritional status of children in food-secure and food-insecure households.	-Socio-economic and demographic questionnaires -HFIAS -HAZ	Household food insecurity was not significantly associated with stunting. 36.9% stunting.	
Betebo, B. et al. [41] 2017 Ethiopia	*N_0_* = 508 6–59 months *N_1_* = 508	The sample is made up of 508 mother−child couples aged 6 to 59 months. The study was conducted in the district of East Badawacho.	Assess household food insecurity and its association with the nutritional status of children aged 6 to 59 months.	-Socio-economic and demographic questionnaires -HFIAS -HAZ	Household food insecurity is significantly associated with stunting. 45.6% stunting.	
Agho, K.E. et al. [42] 2018 Rwanda	*N_0_* = 2222 6–59 months *N_1_* = 660	The present analysis included 2222 children aged 6 to 59 months, conducted in the Gicumbi district.	To examine the association between household food insecurity (HFI) and stunting and severe stunting in children aged 6 to 59 months.	-Sociodemographic questionnaires -HFIAS -HAZ	HFI is significantly associated with stunting. Children aged 24 to 59 months (*p* < 0.001) were more likely to be stunted than children aged 5 to 23 months.	
Shilugu, L.L. et al. [43] 2019 Tanzania	*N_0_* = 358 6–59 months *N_1_* = 358	The sample is made up of 358 tutors and 358 children under 5 years old. This cross-sectional study was carried out in the district of Bukombe.	To examine the extent of stunting among children under 5 in peasant households compared to children from families engaged in other economic activities.	-Sociodemographic questionnaires -24 h reminder -HFIAS -HAZ	Food insecurity is associated with stunting in children, but the association did not reach a statistically significant level (*p* = 0.063). 56.2% stunting.	
Khamis, A.G. et al. [44] 2019 Tanzania	*N_0_* = 2960 6–23 months *N_1_* = 13376	The sample consisted of 13,376 households, and only children aged 6 to 23 months during the study, matched with their mothers, were ultimately selected for further analysis.	Examine the role of children’s dietary diversity on undernutrition in Tanzania using the large dataset available representing the country.	-Sociodemographic questionnaires -24 h reminder -HAZ		There is a significant relationship between dietary diversity and stunting. 31% stunting.
Dinku, A.M. et al. [45] 2020 Ethiopia	*N_0_* = 512 6–59 months *N_1_* = 512	The sample size is 512 mother−child pairs. The study was conducted in the towns of Dessie and Combolcha.	Explore the nutritional status of children aged 6 to 59 months and study their potential covariates.	-Socio-economic and demographic questionnaires -HFIAS -24 h reminder -HAZ	Food insecurity was not associated with any of the anthropometric indices. 42.9% stunting.	Dietary diversity was significantly associated with stunting.
Berra, W.G. et al. [46] 2020 Ethiopia	*N_0_* = 525 6–23 months *N_1_* = 525	The sample for this study is made up of 525 households. The study was carried out in the western areas of Oromia.	Determine the associations between household food insecurity and child undernutrition and assess the prevalence of stunting, underweight, and wasting among the targeted children.	-Sociodemographic questionnaires -HFIAS -HAZ	Children living in moderately food-insecure households were significantly associated with stunting. 16.2% stunting.	
Berhane, H.Y. et al. [47] 2020 Ethiopia	*N_0_* = 5822 0–59 months *N_1_* = 5467	The sample is made up of 5467 households and 5822 children under five. This study was conducted on the population of the city of Addis Ababa.	Describe urban preschool children’s diet and nutritional status, emphasizing stratification and the relative importance of socio-economic factors.	-Socio-economic and demographic questionnaires -HFIAS -24 h reminder -HAZ	Stunting was significantly associated with household food insecurity. The risk of stunting is 1.4 times higher in children from severe food-insecure households. 19.6% stunting.	
**South Africa**						
**Author** **[Ref.],** **Year,** **Country**	**No. of Children (*N0*),** **Age,** **No. of Households (*N1*)**	**Sample/Location**	**Study Purpose**	**Methods of HFI, DD Evaluation and Stunting**	**HFI and Stunting**	**DDS and Stunting**
Faber, M. et al. [48] 2009 South Africa	*N_0_* = 167 6–71 months *N_1_* = 499	The sample includes 499 households. Study was conducted in the Grand Sekhukhune district of Limpopo province.	Describe the relationship between dietary diversity and other indicators of food security.	-Sociodemographic questionnaires -HFIAS -24 h reminder -HAZ	An inverse correlation between HFIAS and dietary diversity (r = 0.450; *p* < 0.01) was observed. 34.7% of the children were stunted.	Households with at least one stunted child had a lower dietary diversity. Dietary diversity is a promising indicator for food security.
Chakona, G. et al. [49] 2017 South Africa	*N_0_* = 216 24–59 months *N_1_* = 554	The study was carried out in three South African cities (Richards Bay, Dundee, and Harrismith). The sample is made up of 554 household women and 216 children from 2 to 5 years old.	Measure and explore the links between household food insecurity, dietary diversity, and children’s nutritional status with the local context.	-Sociodemographic questionnaires -HFIAS -48 h reminder -HAZ	Stunting was significantly associated with access to food as measured by HFIAS for the sample (*p* < 0.02).	The study also showed that access to food household income is positively correlated with stunting. 35% stunting.
Modjadji, P. et al. [50] 2020 South Africa	*N_0_* = 379 36–59 months *N_1_* = 379	The sample includes 379 children aged three to five. The study was conducted in the North West province of South Africa, more specifically in the district of Dr Kenneth Kaunda.	Quantify nutritional status and dietary diversity scores and determine their association in preschool children.	-Sociodemographic questionnaires -24 h reminder -HAZ		No significant association was observed between nutritional indicators and DDS. 29% stunting.

HFI: Household food insecurity, DD: Dietary Diversity, DDS: Dietary Diversity Score, HAZ: Height-For-Age, HFIAS: Household Food Insecurity Access Scale.

## 3. Results

We identified a total 2398 original articles in six databases. After deletion of the duplicates, 1980 articles were retained. A title-based selection resulted in the exclusion of 1865 articles. Abstracts of the remaining 115 articles were read and reviewed, excluding 75 more articles. After reading the full text of the remaining 40 articles, 21 articles were excluded. Thus, 19 articles meeting the inclusion criteria were selected.

Subsequently, a reverse search was performed on the 19 articles included by searching for the terms in the bibliographic references of the studies to identify original published articles that were not initially selected and which met the integration criteria. Two studies were identified and included, totaling 21 articles for this review.

**Figure 1:**
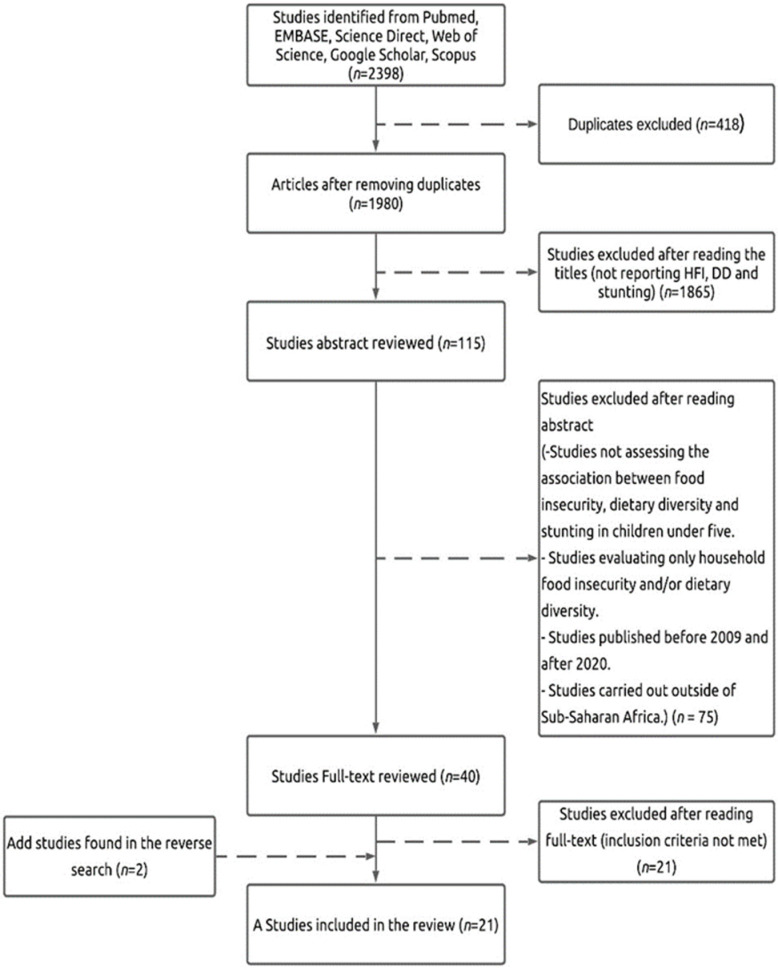
Flowchart for the selection of studies based on the PRISMA 2015 guidelines.

The representative schema of the research and the number of eligible studies are shown in Figure 1.

### 3.1. Characteristics of Included Studies

The studies included in this systematic review have been summarized in Table 1 and grouped by sub-region. Thus, thirteen studies were carried out in East Africa, three in South Africa, three in West Africa, and one in Central Africa. The sample size ranged from 281 to 6552 households and 251 to 6858 children ranging from 6–59 months. A total of 14 studies reported household food insecurity associated with stunting, 12 studies reported dietary diversity related to stunting, 21 studies reported stunting, 12 studies reported dietary diversity, and 14 studies reported household food insecurity.

During the search, one cohort study was found. This cohort study was conducted in Ethiopia [51]. As our systematic review focuses only on cross-sectional studies, the only cohort study was therefore not included.

### 3.2. Evidence from Reviewed Studies

Table 1 shows the relationship between household food insecurity and stunting and the relationship between dietary diversity and stunting. This table also presents the purposes of studies and the methodology used to evaluate these factors.

Concerning the relationship between household food insecurity and stunting and to have a precise assessment, studies using the HFIAS scale were retained (Table 1). Thus, the studies reviewed had indicated that food insecurity was significantly associated with growth retardation [31,36,38,39,41,42,43,46,47,48,49,50], with only some studies [38,40,45] finding that this association was not significant. Moreover, the analysis of this association showed that the proportion of children suffering from stunted growth increases with the level of food insecurity in households [37,39,40,41,46,47] and even more in poor households in food insecurity because food-insecure households generally had lower socioeconomic status than food-secure households [31,39].

In most of the studies reviewed (Table 1), dietary diversity was a factor that significantly increased the risk of stunting in children [33,50], and only two studies [30,32,34,35,36,38,44,45,48,49] did not demonstrate this association. These results also showed that the many of children with a high dietary diversity were less likely to suffer from stunting.

In virtually all of the studies reviewed (n = 20), the prevalence of stunting was reported. Thus, most stunting in children ranged from 23.1% [32] to 78.86% [33]. In addition to the factors listed above, the articles reviewed showed that marital status (married) was also a factor associated with stunting [35,39,42,45], but this association was not statistically significant. However, over 90% of households were married [37,38,40,41,43,44,47,48,49], and only a study carried out in the North West province of South Africa showed the opposite (80% were single people) [52]. At the same time, some studies said the severity of stunting was higher in the younger age group [30,45].

Moreover, the proportion of children with stunting was higher among mothers with less education (primary or lower) [37,39,47] because a greater proportion of mothers in food-insecure households had no formal education. Some studies showed the proportion of boys suffering from stunting is higher than that of girls [36,46], while others showed the opposite [38,40]. Children aged 6 to 59 months from households without year-round access to safe water were more likely to be stunted than those with the ability to access year-round water [31,41].

## 4. Discussion

In our systematic review, household food insecurity was associated with stunting in almost all the articles reviewed for this study. Food insecurity was significantly associated with stunting.

Several similar studies have reported the relationship between household food insecurity and stunting in Canada, Brazil, and China [52,53,54]. In China, this association was important, and the lack of accessibility to food caused by household food insecurity could be a possible cause of stunting in left-behind children [54]. In Brazil, it was also statistically significant and higher for couples whose heads of household had a low level of education [52]. Other studies in Nepal, Thailand, and Cambodia found no association between household food insecurity and stunting [52,55,56]. The proportion of stunted children increased with the level of household food insecurity and even more in poor households. These results show the impact of food security and household wealth on the occurrence of stunting.

In this review, dietary diversity was also significantly associated with stunting in almost all of the studies. Thus, the results of our review are similar to those of Cambodian research, which had shown a significant association between dietary diversity in children and growth retardation in children from 12 to 59 months [55]. In addition, our results were similar to another study conducted in forty-two counties in Western China [57]. Another study conducted in India showed that dietary diversity was an important risk factor for growth retardation in infants and young children [58]. However, these results contrast with the conclusions of other studies which found no association between household dietary diversity and stunting in children under 5 in Cambodia [55], Bangladesh [59], and Colombia [60].

In this study, the analysis of our results revealed that in two thirds of articles selected, household food insecurity and dietary diversity were significantly associated with stunting. Thus, we find that these two factors were significant predictors of stunting. These results show the importance of improving household food security and dietary diversity in order to protect the nutritional status of children. However, this does not exclude the need to better understand the roles of other risk factors. Therefore, it is probable that other determinants could affect the influence of dietary diversity on the nutritional status of children.

Thus, parent’s educational levels associated with stunting are identical to those of another study conducted in Sub-Saharan Africa [61]. The proportion of children with stunting was higher among mothers with a lower level of education (primary or more down) [36,38,46].

However, stunting in children is also linked to the parent’s level of education (mother and father). Likewise, the high level of parental education of parents translates in most cases into higher household income and food security according to a Sub-Saharan Africa study [61]. In Indonesia, a child with a well-educated mother reduces the likelihood of being stunted [62]. In this review, the sex of the child was also linked to stunting. However, the number of boys stunting was higher than of girls [36,46], so girls were less likely to be stunted than boys [38,40]. These results are similar to those found in China and Indonesia [62,63].

This study also demonstrated that marital status was associated with growth retardation, but this association was not significant. These results are similar to those of a study in Papua New Guinea [64] which also found a non-significant association between stunting and marital status. Another study carried out in 31 SSA countries had reached the same conclusion [65]. However, an Indian study had shown that the most significant predictor of stunting was the mother’s age at marriage [66], and a study carried out in five South Asian countries found that children of formerly married mothers were less likely to be stunted than those of currently married mothers [67].

## 5. Recommendations and Strategies to Adopt

This systematic review will help public health researchers and policy-makers to take action and make policy decisions in the fight to reduce malnutrition in SSA. Thus, the recommendations and strategies to be taken would be, among other things, to improve food security through diversification and availability of food for low-income households. These actions should also concern the education of women and girls, improvement of access to drinking water, modification of access to care through better use of maternal and child health services, and the fight against poverty by improving the socio-economic status of households.

## 6. Strengths and Limitations

We conducted an in-depth search of the existing literature on the association between household food insecurity, dietary diversity, and stunting in SSA. To our knowledge, this is the first review to identify the association between these three factors. All the studies included were of high quality.

Several limitations are present in this study. First, qualitative analysis studies were among the exclusion criteria in this review, and only quantitative cross-sectional studies were retained. The inclusion of qualitative studies in systematic reviews allows triangulation of results or offers alternative explanations [29]. Second, we excluded case studies, books, policy briefs, or theses. Finally, this systematic review of transversal design is limited in its capacity to establish a causal relationship between stunting, dietary diversity, and household food insecurity.

## 7. Conclusions

This study showed that household food insecurity and dietary diversity were significantly associated with stunting in Sub-Saharan Africa. However, this review also found that the parent’s marital status (married) is a common factor associated with stunting even though this association was not significant. Therefore, there is a need to implement reliable guidelines and strategies to improve household food security and combat child malnutrition. These approaches should include counseling sessions for parents to improve household food security, training sessions for mothers to improve breastfeeding practices and maternal nutrition, and awareness campaigns for good hygiene and sanitation practices. These strategies will make it possible to fight malnutrition effectively and sustainably in the countries of Sub-Saharan Africa.

## Supplementary Materials

The following are available online at https://www.mdpi.com/article/10.3390/nu13124401/s1, Table S1: Risk of Bias of the Selected Studies by JBI-MAStARI.
