# Supplementary material for: Household Food Insecurity, Dietary Diversity, and Stunting in Sub-Saharan Africa: A Systematic Review
Source: Nutrients. 2021 Dec 9;13(12):4401. doi: 10.3390/nu13124401 (PMC8707760; doi:10.3390/nu13124401)
Supplement: Supplementary file 1 [file nutrients-13-04401-s001.zip › nutrients-1461089-supplementary.pdf]

## Supplementary Data

**Supplementary Table S1:** Risk of Bias of the Selected Studies by JBI-MAStARI

| Author, Year, Ref | Q1 | Q2 | Q3 | Q4 | Q5 | Q6 | Q7 | Q8 | Yes Score (_/8) Methodological quality |
|---|---|---|---|---|---|---|---|---|---|
| Hatley A et al. 2000 [30] | Y | Y | Y | Y | Y | Y | N | Y | 7/8 |
| Mahama S et al. 2013 [31] | Y | Y | Y | Y | Y | Y | Y | Y | 8/8 |
| Ali S et al. 2018 [32] | Y | Y | Y | Y | Y | Y | N | Y | 7/8 |
| BN Ekesa et al. 2011 [33] | Y | Y | Y | Y | Y | Y | N | Y | 7/8 |
| Ali D et al. 2013 [34] | Y | Y | Y | Y | Y | Y | Y | Y | 8/8 |
| Zipporah N et al. 2014 [35] | Y | Y | Y | Y | Y | Y | N | Y | 7/8 |
| Motbainor A et al. 2015 [36] | Y | Y | Y | Y | Y | Y | Y | Y | 8/8 |
| Mutisya M et al. 2015 [37] | Y | Y | Y | Y | Y | Y | N | Y | 7/8 |
| M'Kaibi FK et al. 2016 [38] | Y | Y | Y | Y | Y | Y | Y | Y | 8/8 |
| Abdurahman AA et al. 2016 [39] | Y | Y | Y | Y | Y | Y | Y | Y | 8/8 |
| Mulu et al. 2017 [40] | Y | Y | Y | Y | Y | Y | Y | Y | 8/8 |
| Betebo B et al. 2017 [41] | Y | Y | Y | Y | Y | Y | Y | Y | 8/8 |
| Agho KE et al. 2018 [42] | Y | Y | Y | Y | Y | Y | Y | Y | 8/8 |
| Shilugu LL, et al. 2019 [43] | Y | Y | Y | Y | Y | Y | Y | Y | 8/8 |
| Khamis AG et al. 2019 [44] | Y | Y | Y | Y | Y | Y | Y | Y | 8/8 |
| Dinku AM et al. 2020 [45] | Y | Y | Y | Y | Y | Y | Y | Y | 8/8 |
| Berra WG et al. 2020 [46] | Y | Y | Y | Y | Y | Y | Y | Y | 8/8 |
| Berhane HY et al. 2020 [47] | Y | Y | Y | Y | Y | Y | Y | Y | 8/8 |
| Faber M et al. 2009 [48] | Y | Y | Y | Y | Y | Y | Y | Y | 8/8 |
| Chakona G et al. 2017 [49] | Y | Y | Y | Y | Y | Y | Y | Y | 8/8 |
| Modjadji P et al. 2020 [50] | Y | Y | Y | Y | Y | Y | Y | Y | 8/8 |

The items were collapsed into 8 quality-appraisal criteria (Q1-Were the criteria for inclusion in the sample clearly defined? Q2-Were the study subjects and the setting described in detail? Q3-Was the exposure measured in a valid and reliable way? Q4-Were objective standard criteria used for measurement of the condition? Q5-Were confounding factors identified? Q6-Were strategies to deal with confounding factors stated? Q7-Were the outcomes measured in a valid and reliable way? Q8-Was appropriate statistical analysis used?).

JBI-MAStARI was used to assess risk of bias. Articles that scored between 1 and 2 were classified as low methodological quality, articles with scores between 3 and 4 were classified as moderate quality, and those with scores > 5 were classified as high quality.

N, no; NA, not applicable; U, unclear; Y, yes.
